# Tissue distribution of emulsified γ-tocotrienol and its long-term biological effects after subcutaneous administration

**DOI:** 10.1186/1476-511X-13-66

**Published:** 2014-04-09

**Authors:** Lili Deng, Ying Peng, Yu Wu, Meilin Yang, Yuedi Ding, Quancheng Chen, Qiang Fu

**Affiliations:** 1 Jiangsu Institute of Nuclear Medicine, QianRong Road #20, Wuxi, Jiangsu 214063, China; 2 The First Affiliated Hospital of Nanjing Medical University, Nanjing, Jiangsu 210029, China

## Abstract

**Background:**

γ-tocotrienol (GT3), an analogue of vitamin E, has gained increasing scientific interest recently as it provides significant health benefits. It has been shown that emulsified GT3, after subcutaneous administration, has long-term biological effects. However, whether the effects are due to the increase of GT3 level in the early phase following administration or the persistent functions after accumulation in tissues is unknown. This study was conducted to determine the levels of GT3 in different tissues by high performance liquid chromatography (HPLC) with a fluorescence detector after a single-dose of GT3 with polyethylene glycol (PEG-400) emulsion via subcutaneous injection. Previous studies have explored that GT3 has favorable effects on bone and can inhibit osteoclast formation. To confirm the persistent biological activity of accumulated GT3 in tissues, receptor activator of NF-κB ligand (RANKL) and osteoprotegerin (OPG) gene expressions, which have an important role in regulating osteoclast formation, were also evaluated in bone tissue on day 1, 3, 7 and 14 after a signal subcutaneous injection of GT3.

**Methods:**

C57BL/6 female mice were administrated GT3 (100 mg/kg body weight) with PEG-400 emulsion by subcutaneous injection. GT3 levels in different tissues were determined by HPLC with a fluorescence detector. Gene expressions were measured by real-time PCR.

**Results:**

GT3 predominantly accumulated in adipose and heart tissue, and was maintained at a relatively stable level in bone tissues after a single-dose administration. Accumulated GT3 in bone tissues significantly inhibited the increase in RANKL expression and the decrease in OPG expression induced by db-cAMP.

**Conclusions:**

We investigated the tissue distribution of GT3 with PEG emulsion by subcutaneous administration, which has never been reported so far. Our results suggest that GT3 with PEG emulsion accumulated in tissues is able to carry out a long-term biological effect and has therapeutic value for treating and preventing osteoporosis.

## Background

Vitamin E, one of the essential micronutrients and a known antioxidant, includes two groups of closely related fat-soluble compounds, the tocopherols and tocotrienols, each with the four analogues, α, β, γ, and δ. These isoforms differ in the saturation of their side chains and both the number of methyl substituents and their positions in the chromanol ring [1,2].

For the most part, early antioxidant studies focused on α-tocopherol, the predominant form of vitamin E existing in human and animal tissues. Based on epidemiologic and retrospective studies, supplementation with Vitamin E (mainly α-tocopherol) is considered to be a nutrition-based strategy to prevent diseases and to support healthy aging. However, a new study in mice showed that α-tocopherol can stimulate bone osteoclast fusion independent of its antioxidant activity, resulting in increased bone resorption [3]. The results from that study raise a question of whether vitamin E (mainly α-tocopherol) taken as dietary supplement is suitable considering it may have a risk of osteoporosis.

On the other hand, tocotrienols have gained increasing scientific interest during recent years as they have been reported to possess certain biology activities that were not observed in tocopherols [4]. This is especially true for γ-tocotrienol (GT3), which is abundant in palm oil and rice bran [5,6], as it provides significant health benefits, including anticancer [7-10], anticholesterolemic [11,12], antihypertensive [13], and antiatherosclerotic effects [14], as well as acting as a potent antioxidant [15,16]. Moreover, GT3 is a particularly potent radio-prophylactic agent in vivo [17] and has favorable effects on bone [18]. All of these studies have shown γ-tocotrienol health benefits to be significantly greater than those of α-tocopherol.

Despite the growing interest in GT3, there is a paucity of information about bioavailability and there is still much controversy about its absorption, retention, and metabolism. α-tocopherol is preferentially incorporated into very low-density lipoproteins and transported to various tissues by lipoprotein because of its high affinity for α-tocopherol transfer protein (α-TTP) [19]. In contrast, the absorption of tocotrienols has been found to be low and incomplete by oral administration [20]. The presence of a transfer protein that preferentially selects α-tocopherol seems to explain why all other forms of vitamin E have a lower biological activity compared with α-tocopherol. Therefore, oral administration is unsuitable for GT3 biological activity studies in vivo.

In the previous studies of radioprotection, we found that administration of a single-dose of GT3 with polyethylene glycol (PEG-400) emulsion by subcutaneous injection could ameliorate radiation injury, including radiation-induced vascular oxidative stress, gastrointestinal injury, protection of hematopoietic stem and progenitor cells depletion, and induction of cytokines production. These effects led to enhancement of hematopoietic recovery and improvement of 30-day survival after total body irradiation (TBI) [21-23]. These results suggested that administration of emulsified GT3 by subcutaneous injection has long-term biological effects; however, whether the biological effects are due to the increase of GT3 level in the early phase or the persistent functions as the accumulation in tissues is unknown. Therefore, it is necessary to investigate the biodistribution of emulsified GT3 and the duration of biological activity in vivo after a single-dose of GT3 administration.

Receptor activator of NF-κB ligand (RANKL) is a member of the tumor necrosis factor (TNF) family of cytokines and is expressed by stromal/osteoblast, hypertrophic chondrocytes, osteocytes and other cell types, including T and B lymphocytes [24]. Binding of RANKL to its receptor RANK triggers the activation of multiple downstream signaling which regulates osteoclast formation, activation and survival in normal bone modeling and remodeling [25]. Osteoprotegerin (OPG) is a soluble decoy receptor for RANKL that blocks ligand binding to RANK, thereby preventing the signaling required for osteoclast differentiation and activation [26]. The relative concentration of RANKL and OPG in bone is a major determinant of bone mass and the RANKL/RANK/OPG system is critical for skeletal health, disruption of this system leads to or causes numerous bone diseases [27]. Previous studies have reported that γ-tocotrienol can inhibit osteoclast formation and improve normal bone structure [28,29].

In this study, we determined the durative levels of GT3 in different tissues by high performance liquid chromatography (HPLC) with a fluorescence detector after administration of a single-dose of GT3 with polyethylene glycol (PEG-400) emulsion by subcutaneous injection. In addition, we also investigated the persistent biological activity of accumulated GT3 by examining RANKL and OPG gene expression in bone tissues at various time intervals following db-cAMP administration.

## Results

Previous studies have detected GT3 levels by oral administration or dietary intake [30-32]. The GT3 levels in tissues of SD rats by intragastric administration has also been reported [33]. However, the distribution of GT3 in various tissues by subcutaneous administration has not been investigated. In the present study, we determined the tissue distribution of GT3 via this administration route by high performance liquid chromatography (HPLC).

Silica normal-phase column chromatography can separate all eight tocopherols and tocotrienols and is the most available HPLC method for GT3 detection in plants and food which originated from plants [6,34,35]. However, octadecylsilyl (ODS) columns are widely used for LC separation; and reversed-phase methods are usually preferred over the normal-phase methods. As GT3 is the sole vitamin E analogue in our experimental samples, a reversed-phase HPLC method with a fluorescence detector was utilized for GT3 analysis in our study.

**Figure 1:**
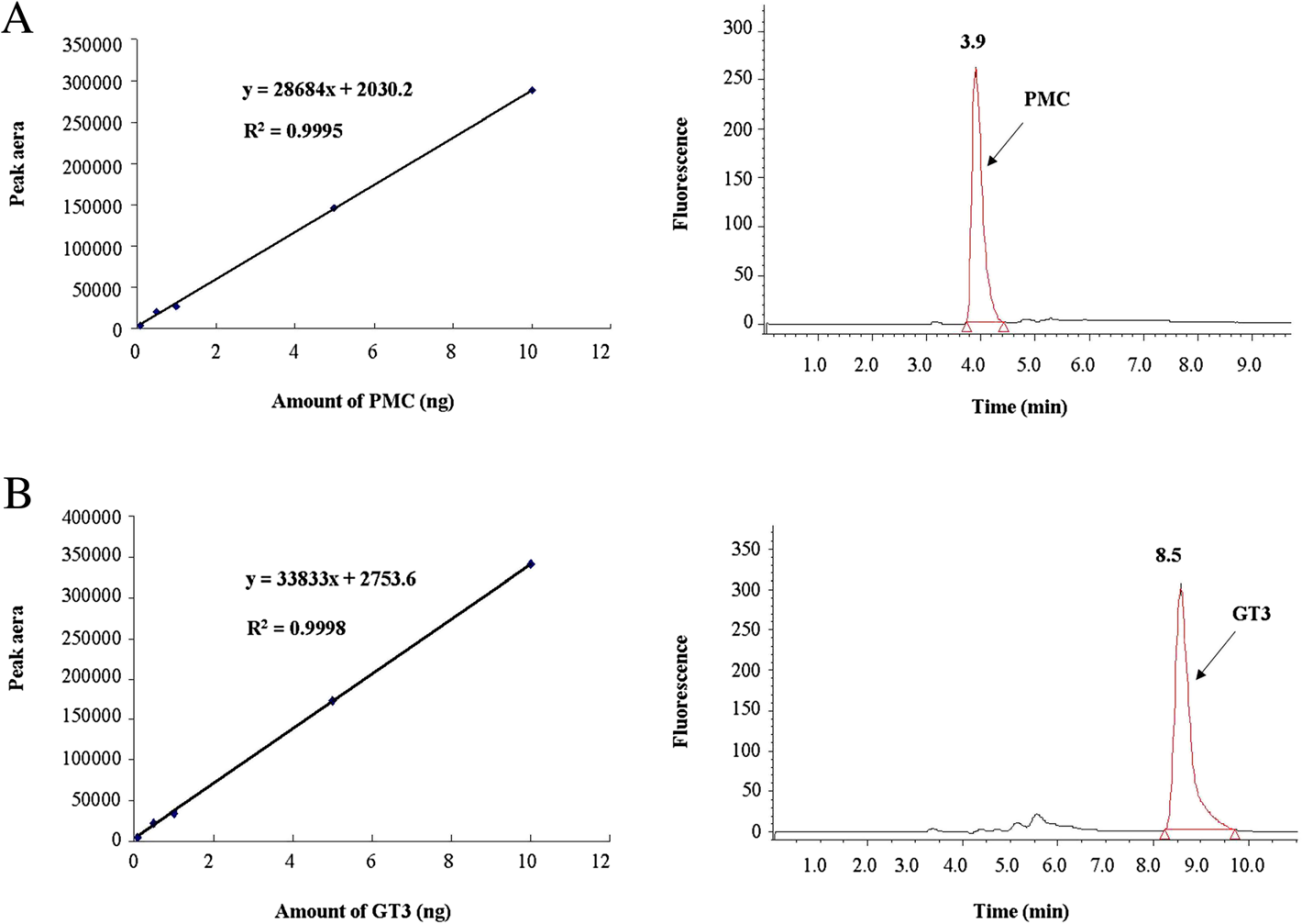
**Calibration curves and chromatograms.** HPLC calibration curves and chromatograms of PMC **(A)** and GT3 standard sample **(B)**.

In order to calculate the recovery of extraction of GT3, 2,2,5,7,8-pentamethyl-6-chroman (PMC) was used as an internal standard as introduced by Ueda and Igarashi [36]. The calibration curves of both GT3 and PMC were linear in the range with correlation coefficients (r) larger than 0.999 and regression equations of y = 33833x + 2753.6 and y = 28684x + 2030.2 for GT3 and PMC, respectively (Figure 1). The detection limit of quantification was 0.1 ng, which represents the lowest concentration in the calibration curves. Under the chromatographic conditions described above, PMC and GT3 was eluted at 3.9 and 8.5 min, respectively. Since both PMC and GT3 were eluted within a short time, the analytical run time was optimized to 12 min, which is shorter than those of normal-phase HPLC methods with a run time of at least 20 min [6,34,35]. Therefore, our results suggested that the reversed-phase HPLC method was simple and fast, making it applicable for routine analysis of GT3 in tissues.

**Figure 2:**
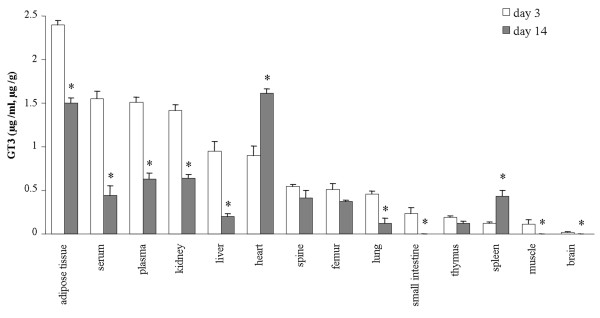
**Histogram of tissue distribution of GT3.** Histogram of tissue distribution of GT3 on day 3 and 14 after subcutaneous injection of GT3 with PEG-400 emulsion. The level of control group was used as a baseline value and the concentrations of GT3 were subtracted from baseline value for the experiment groups (day 3 and day 14). *, P < 0.05 by Student’s t test compared with day 3.

GT3 levels in all examined tissues, including plasma and serum were detected on day 3 and 14 after subcutaneous injection of GT3 with PEG-400 emulsion. Previous studies that detected GT3 levels after oral administration or dietary intake in rats, mice, and hamsters have shown that GT3 specifically accumulated in skin and adipose tissue [30-32]. In our study, GT3 was predominant in adipose tissue on day 3 and was still higher on day 14 after administration of a single-dose of emulsified GT3 by subcutaneous injection (Figure 2). This is consistent with the studies in which GT3 was administered in the diet, indicating that GT3 predominantly accumulates in adipose tissue no matter what routes of administration were used. It is interesting to find that GT3 levels in heart and spleen significantly increased on day 14 compared to the level on day 3 (Figure 2). The increasing levels of GT3 in these tissues may be due to these two organs having the advantage to re-uptake GT3 from plasma after release from adipose tissue. Actually, as a radioprotector, GT3 has strong beneficial effects on the cardiovascular system and spleen colony formation [22]. For bone tissues (femur and spine), the GT3 level was relatively stable and had no significant change (*P* > 0.05) from day 3 to day 14 after administration (Figure 2).

Currently, there is still limited information available regarding the metabolism of tocotrienols. Some studies report that dietary tocotrienols are absorbed in the small intestine and secreted with triacylglycerol-rich chylomicrons into the lymph and blood; then after that, the absorbed tocotrienols are transported to the liver [37,38]. Our results indicate that GT3 levels in the liver are relatively lower than in the heart, kidney and adipose tissue, particularly on day 14 after subcutaneous delivery, implying a possibility of liver-independent transport of GT3 into the various tissues after subcutaneous injection.

**Figure 3:**
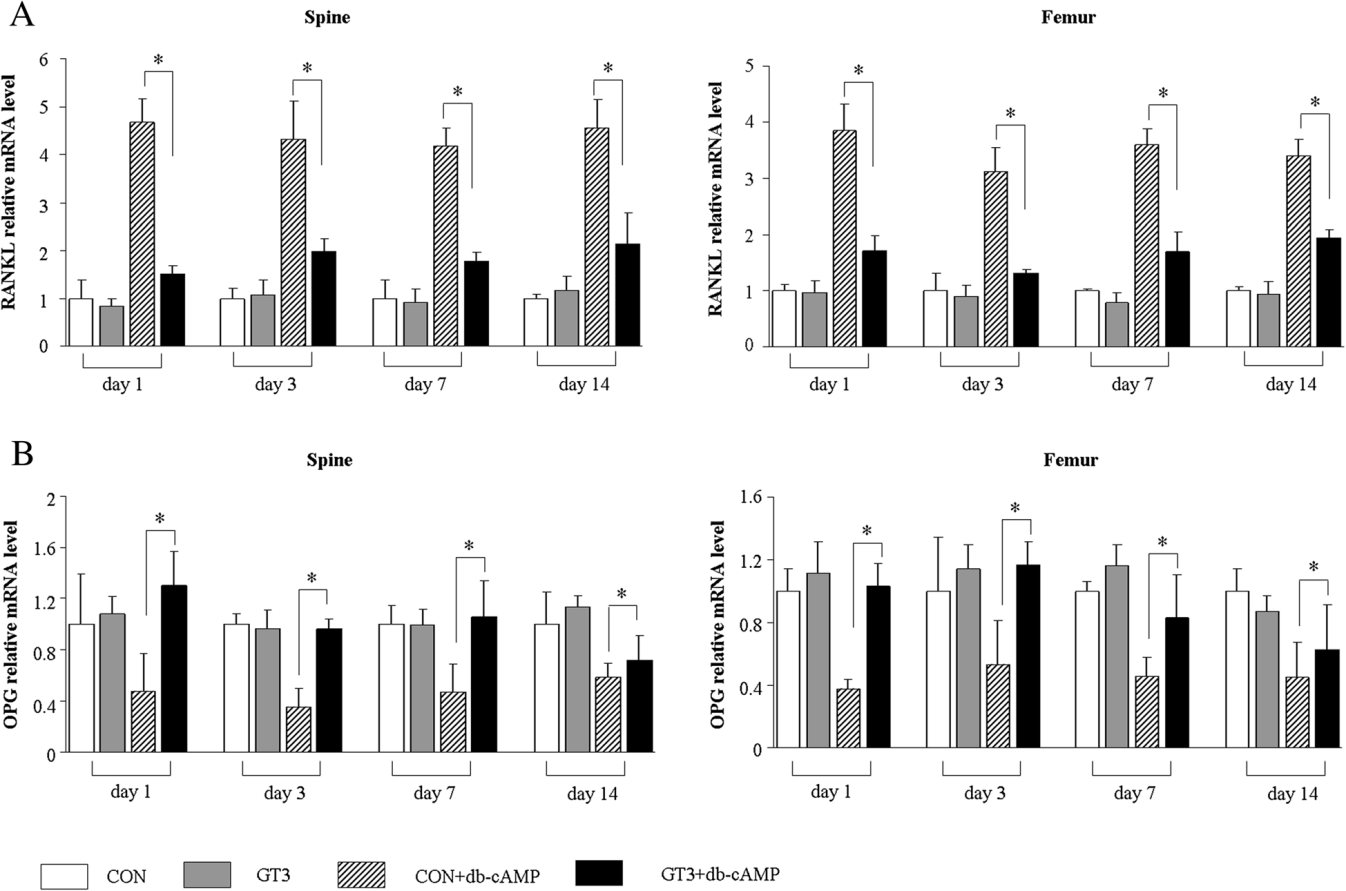
**GT3 on RANKL and OPG gene expression.** The effect of accumulated GT3 on expression of RANKL **(A)** and OPG **(B)** gene stimulated by db-cAMP in bone tissues (spine and femur). Excipent (CON and CON + db-cAMP groups) or GT3 (100 mg/kg body weight, GT3 and GT3+ db-cAMP groups) with PEG-400 emulsion were subcutaneously injected on day 0. One hour before mice were sacrificed at the time point of the indicated in figures, mice were injected intraperitoneally with db-cAMP (100 mg/kg body weight, CON + db-cAMP and G + db-cAMP groups) in a total volume of 100 μL or phosphate-buffered saline (PBS, CON and GT3 groups). Expression in other groups was normalized to expression in CON group. Each bar represents the mean ± STDEV of eight mice. *, *P* < 0.05 by one-way ANOVA followed by Tukey’s post hoc test.

GT3 with PEG emulsion after subcutaneous administration has long-term biological effects (20–22); however, it is not clear whether the durative biological effects are the result of the rapid increase in GT3 level in the early phase after administration or the accumulation of GT3 in tissues. As GT3 level was relatively stable in bone tissues, to investigate whether accumulated GT3 still had biological effects after a period of time, RANKL and OPG mRNA expression were examined. RANKL and OPG are both produced by osteoblastic cells and are indicative of osteoclastogenesis stimulation and inhibition respectively. In vivo, RANKL expression is up-regulated in bone and lymphoid tissues after administration of db-cAMP, a cell permeable analogue of cAMP [39]. Our results showed that RANKL was increased and OPG was decreased in mRNA levels after administration of db-cAMP; accumulated GT3 in bone tissues inhibited RANKL expression and blocked the down-regulation of OPG, even on day 14 after the single-dose of emulsified GT3 supplementation (Figure 3). Although the reduction of OPG expression on day 14 appeared to lessen, the data still suggested that emulsified GT3 has persistent biological effects after accumulation in tissues.

A growing number of studies have shown that GT3 has more potent antioxidative, anti-hypercholesterolemic, and anticancer than tocopherols, suggesting that GT3 has significant health benefits. However, the absorption of tocotrienols was found to be low and incomplete via the oral route and only approximately 9% bioavailability can be observed in vivo [20]. Therefore, it is necessary to explore other methods of administration to improve biological activity in vivo. Fat soluble vitamins including GT3 are better absorbed in the presence of surfactants or from emulsified vehicles. Thus, there is increased interest in the use of self-emulsifying systems in which lipophilic drugs such as GT3 may be stored in concentrated oil-surfactant solution to be reconstituted with water to form an emulsion immediately before use [40]. In our study, we utilized polyethylene glycol (PEG-400) as the emulsifier and such self-emulsifying system has been approved that can achieve a faster rate and higher extent of absorption.

## Conclusions

In the present study, we investigated the tissue distribution of GT3 (packed with PEG emulsion) after subcutaneous administration, which has never been reported so far. We found that GT3 predominantly accumulated in adipose tissue and heart; and was maintained at a relatively stable level in bone tissues after a single-dose administration. Furthermore, we also demonstrated that emulsified GT3 has a long-term biological activity after accumulation in tissues via subcutaneous injection. GT3 inhibited RANKL up-regulation and blocked OPG down-regulation which prevents bone loss. These results suggested that emulsified GT3 has significant health benefits and may have therapeutic value for treating and preventing osteoporosis.

## Methods

### Materials

GT3 (a kind gift of M. Hauer-Jensen) was obtained from Yasoo Health Inc. (Johnson City, TN). Shortly before administration, GT3 was dispersed in a mixture of PEG-400 (Sigma, St. Louis, MO). 2,2,5,7,8-pentamethyl-6-chroman (PMC) as an internal standard for HPLC was purchased from Sigma. Hexane (Tedia Company Inc, USA), alcohol (Tedia Company Inc, USA), methanol (Merk KGaA, Germany), acetonitrile (Tedia Company Inc, USA) and methylene chloride (Merk KGaA, Germany) were of HPLC grade. Dibutyryl-cAMP (db-cAMP) was purchased from Sigma. All other reagents and chemicals were commercially available extra-pure-grade products.

### Animals

The experimental protocol was reviewed and approved by the Institutional Animal Care and Use Committees (IACUC) of Jiangsu Institute of Nuclear Medicine (JSINM2010007).

C57BL/6 female mice (Eight-week-old, about 23 ~ 25 g) were purchased from SLAC laboratory animals Lit. (Shanghai, China). They were housed under standardized conditions with controlled temperature and humidity and a 12-12-h day-night light cycle. Mice were given free access to a vitamin E-free diet and water.

### Tissue distribution of GT3 by HPLC

After acclimatizing for one week, mice were administrated GT3 (100 mg/kg body weight) with PEG-400 emulsion or excipient (olive oil) by subcutaneous injection. Eight mice for each treatment group were euthanized on day 3 and 14 after administration of emulsified GT3 or excipient. Blood samples were collected into heparinized tubes and directly centrifuged at 5000 × g for 10 min to separate the plasma or collected into normal tubes to separate serum. After blood collection, the heart, liver, spleen, lung, kidney, thymus, small intestine, brain, spine, femur, muscle, and adipose tissue were immediately harvested. All samples were stored at −80 °C until analysis.

GT3 was extracted by following method. For blood samples, 500 μL plasma or serum was added to test tubes containing 500 μL SDS (0.05 M), 1 mL alcohol (90.2% ethanol, 4.9% methanol, and 4.9% isopropanol), and 0.01 g pyrogallol with 1 μg 2,2,5,7,8-pentamethyl-6-chroman (PMC) as an internal standard. Next, 2 mL of hexane was added to the mixture and vortex-mixed thoroughly. The mixture was then centrifuged at 4000 × g for 10 min. For other tissues, about 0.1 g of each sample was homogenized with 2 mL alcohol containing 0.02 g pyrogallol and 1 μg PMC. Then, 200 μL potassium hydroxide (800 g/L) was added and incubated in 70°C water bath for 30 min. After saponification, the tubes were placed on ice for 5 min, followed by the addition of 2 mL hexane and shaken vigorously for 2 min and then centrifuged at 1500 × g for 10 min. After drying the supernatant under an N_2_ gas flow, the residue was dissolved in 100 μL ethanol.

The instrument used for HPLC was a Waters 6000 Controller with Waters 2475 multi λ fluorescence detector (excitation 298 nm, emission 325 nm). The analytical column used was a C18 reverse-phase column (250 × 4.6 mm, Inertsil, ODS-3; GL Science). The mobile phase consisted of methanol, acetonitrile and methylene chloride (50: 44: 6, v/v/v) with a flow rate of 1 mL/min. The injection volume was 10 μL.

### Gene expression analysis

Mice received a single-dose of GT3 (100 mg/kg body weight) with PEG-400 emulsion (GT3 group) or excipient alone (vehicle group) by subcutaneous injection. One hour before being sacrificed, both GT3 and vehicle group mice were injected intraperitoneally with db-cAMP (100 mg/kg body weight) in a total volume of 100 μL or phosphate-buffered saline (PBS) alone as control. Eight mice were sacrificed for each group on day 1, 3, 7 and 14; spine and femur were harvested. The expressions of RANKL and OPG induced by db-cAMP were measured by real-time PCR.

Total RNA was purified by using Ultraspec reagent (Biotecx Laboratories, Houston, TX) according to the manufacturer’s instructions. Murine RANKL, OPG and the housekeeping gene ribosomal protein S2 were amplified from the first-stand cDNA by using Taqman quantitative PCR master Mix (Applied Biosystems). The TaqMan assay numbers of the primer/probe sets used are: RANKL, Mm00441908m1; OPG, Mm00435452m1; ribosomal protein S2 (forward, 5’-CCCAGGATGGCGACGAT-3’; reverse, 5’-CCGAATGCTGTAATGGCGTAT-3’; probe, FAM-5’-TCCAGAGCAGGATCC-3’-NFQ). PCR amplification and detection were carried out on an ABI Prism 7500 Sequence Detecction System (Applied Biosystems) according to the manufacturer’s instructions. Relative mRNA levels were calculated using the ΔCt method [41]. All mRNA levels are normalized to ribosomal protein S2 mRNA levels.

### Statistical analysis

Data are presented as the group means ± STDEV. A statistical analysis was performed by using Student's t test for two different groups or one-way ANOVA followed by Tukey’s post hoc test for multiple comparisons. *P* < 0.05 is considered to indicate a significant difference. All data were analyzed using SPSS 17.0.

## Competing interests

The authors declare that they have no competing interests.

## Authors’ contributions

LD participated in the design of the study, carried out tissue distribution experiment and drafted the manuscript. YP carried out RANKL and OPG gene expressions and helped to draft the manuscript. YW performed the statistical analysis. MY kept mice and carried out animal experiment. YD extracted RNA and participated in study of gene expressions. QC helped to determine GT3 levels by HPLC. QF conceived of the study, made substantial contributions to the design of this manuscript, and gave final approval of the version to be published. All authors read and approved the final manuscript.
